# Asymptomatic Prenatal Zika Virus Infection and Congenital Zika Syndrome

**DOI:** 10.1093/ofid/ofy073

**Published:** 2018-04-07

**Authors:** Enny S Paixao, Wei-Yee Leong, Laura C Rodrigues, Annelies Wilder-Smith

**Affiliations:** 1 Infectious Disease Epidemiology and Department of Disease Control, London School of Hygiene and Tropical Medicine, London, UK; 2 Lee Kong Chian School of Medicine, Singapore, Singapore; 3 Department of Public Health and Clinical Medicine, Umea University, Umea, Sweden

**Keywords:** asymptomatic prenatal infection, congenital Zika syndrome, maternal infection, microcephaly, Zika

## Abstract

To investigate to what extent asymptomatic vs symptomatic prenatal Zika virus infections contribute to birth defects, we identified 3 prospective and 8 retrospective studies. The ratio varied greatly in the retrospective studies, most likely due to recruitment and recall bias. The prospective studies revealed a ratio of 1:1 for asymptomatic vs symptomatic maternal Zika infections resulting in adverse fetal outcomes.

Ever since the causal relationship between prenatal Zika virus (ZIKV) infection and microcephaly (and other serious brain anomalies) was established, the research focus has been on defining the full spectrum of defects caused by prenatal Zika virus infection, determining the relative and absolute risks of adverse outcomes among fetuses whose mothers were infected at different times during pregnancy and factors associated with adverse fetal outcomes [1]. An additional important research question is whether asymptomatic infections during pregnancy can also lead to congenital Zika syndrome (CZS), and the extent of this risk in comparison with symptomatic prenatal ZIKV infections. Based on a seroprevalence study in Yap Island, an asymptomatic-to-symptomatic ratio of 4:1 has been postulated in the general population [2]. However, this ratio was derived from nonspecific serological assays. Furthermore, such ratios may vary in different settings. For example, for dengue virus infections, another flavivirus transmitted by the same vector, asymptomatic-to-symptomatic ratios depend on age and viral virulence [3]. Pregnancy may also be associated with more clinical manifestations than reported in the general population. Knowing the proportion of asymptomatic ZIKV infection in pregnant women, and the extent to which asymptomatic ZIKV infections lead to birth defects, is critical to understanding the evolving epidemiology, selecting the best diagnostic approach in pregnant women, and informing vaccine development. Setting up sufficiently large prospective cohort studies of pregnant women living or visiting countries during a Zika epidemic will be the best study design to accurately determine these proportions. Indeed, such studies are being funded by the National Institutes of Health/National Institute of Allergy and Infectious Diseases (https://www.nichd.nih.gov/news/releases/Pages/zika_zip_06202016.aspx) and the European Commission [4], but definitive answers will not be able for another couple of years. In the absence of such results, we did a literature review to assess whether asymptomatic ZIKV infection during pregnancy is associated with CZS/microcephaly and to estimate the risk.

## THE STUDY

Using the search terms “Zika” AND/OR “pregnancy” AND/OR “microcephaly,” we identified 898 papers between 1947 and September 2017. We reviewed all titles and abstracts of publications and selected those articles that fulfilled the following eligibility criteria: original articles, birth outcomes from pregnant women with prenatal laboratory-confirmed ZIKV infection where clinical symptoms and the absence of clinical symptoms were reported. Eligible study designs were case series, case-control studies (in which the case was congenital Zika syndrome or microcephaly and presence or absence of symptoms during pregnancy was described), prospective studies following up returning travelers, and cohort studies. We excluded all studies where women were recruited only because of rash or other symptoms. We identified 11 articles published from 2016 to 2017 conducted in 3 countries (Brazil, Colombia, and the United States) that fulfilled our inclusion criteria. Using a standardized tool, we extracted the following information: study design, year of publication, study location, period of study, study population, clinical symptoms in mothers, laboratory confirmation of ZIKV infection in mothers, adverse fetal outcome, and frequency of such outcomes in neonates.

Table 1 shows that the studies can be classified into those that recruit pregnant women and prospectively ascertain fetal outcomes [5–7] and those studies that recruit neonates with CZS or microcephaly and establish retrospectively whether the mother had symptoms compatible with a ZIKV infection [8–15]. The US cohort of pregnant women with laboratory-confirmed ZIKV infections offered the highest quality of evidence as the study was prospective in nature; women were enrolled during pregnancy without prior knowledge about birth outcome, which minimizes recall bias for “Zika-like symptoms” compared with retrospective case-control studies and case series [5–7]. In the 3 prospective studies reporting on 442 [5] to 2549 [7] completed pregnancies, the proportion of symptomatic maternal ZIKV infections ranged from 38% to 61%. In the first report, 6% of asymptomatic and equally 6% of symptomatic maternal ZIKV infections resulted in CZS [5]; in the second report, 5% of women were symptomatic and 4% were asymptomatic [6]; and in the most recent report, 5% of women were symptomatic and 7% were asymptomatic [7]. In other words, the ratio of asymptomatic to symptomatic ZIKV infections resulting in adverse fetal outcomes is about 1:1. Among the completed 2549 pregnancies, there was no difference in the percentage of birth defects (between symptomatic and asymptomatic women) in the subgroup of laboratory-confirmed Zika infection [7].

**Table 1.** Study Characteristics

Author; Year	Study Location	Study Population	Study Design	Sample Size	Main Findings
Prospective studies					
Honein et al; 2017 [5]^a^	USA	Pregnant travelers returning to the US who had acquired laboratory-confirmed ZIKV infection while traveling to a country with ZIKV transmission	Cohort study	442 completed pregnancies	271 (61%) were asymptomatic, 167 (38%) were symptomatic; Birth defects were identified in 26 (6%) fetuses or infants out of the 442 completed pregnancies. Birth defects were reported in 16 (6%) of 271 pregnant asymptomatic women and 10 (6%) of 167 symptomatic pregnant women. Infant brain abnormalities and/or microcephaly were reported in 8 (4.8%) pregnant women with symptomatic and 14 (5.1%) pregnant women with asymptomatic ZIKV infection.
Reynolds et al; 2017 [6]^a^	USA	Pregnant travelers returning to the US who had acquired laboratory-confirmed ZIKV infection while traveling to a country with ZIKV transmission	Cohort study	972 completed pregnancies	Among 250 lab-confirmed maternal ZIKV infection cases, 141 (56.4%) reported symptoms and 102 (40.8%) reported no symptoms. 11/141 (7.8%) symptomatic pregnancies had birth defects compared with 12/102 (11.8%) asymptomatic pregnancies. Out of 972 completed pregnancies, 51 (5%) reported ZIKV-associated birth defects. The proportion was higher among laboratory-confirmed cases, 10% (24/250). Birth defects were reported in a higher proportion of fetuses or infants whose mothers were infected during the first trimester (15%). Live-births: 94 (11%) of all 895 live-born infants had positive Zika virus test results. Among the 45 live-born infants with birth defects, 25 (56%) had a positive Zika virus test reported.
Shapiro-Mendoza et al; 2017 [7]^a^	USA	Pregnant travelers returning to the US who had acquired laboratory-confirmed ZIKV infection while traveling to a country with ZIKV transmission	Cohort study	2549 completed pregnancies	1561 (61%) pregnant women reported signs or symptoms compatible with maternal ZIKV infection, and 966 (38%) were asymptomatic. Among the 2549 completed pregnancies, 122 (5%) resulted in a fetus or infant with possible ZIKV-associated birth defects (5% among symptomatic and 4% among asymptomatic women). The percentages of CZS among 1508 pregnancies with NAT-confirmed ZIKV infection were 5% among symptomatic and 7% among asymptomatic women.
Retrospective studies					
Araujo et al; 2016 [8]	Brazil	Cases: neonates with microcephaly; controls: neonates without microcephaly	Case-control	32 cases and 62 controls	Mothers of the cases: 24/30 (80%); 39/61 (64%) mothers of controls had laboratory-confirmed ZIKV via PRNT; 19/32 (59%) mothers of cases vs 46/62 (74%) controls reported no rash (asymptomatic). 13/32 (41%) cases of neonates were either ZIKV positive by RT-PCR or IgM (serum or CSF); out of these 13 neonates with lab-confirmed ZIKV infection, 7 (53.8%) of the mothers reported rash (symptomatic). None of the neonates in control group were positive for ZIKV testing.
Oliveira-Szejnfeld et al; 2016 [9]	Brazil	Pregnant women or fetuses with abnormalities	Case series	16 confirmed ZIKV cases in neonates	In the 16 neonates whose mothers had confirmed ZIKV infection during pregnancy, rash was reported in 13 (81%) in the first trimester.
Aragao et al; 2017 [10]	Brazil	Infants who had brain MR and CT scans at age 1 year or younger in 1 specific center from 2015–2016	Case series	77 infants with brain scans (CT/MR)	Out of the 77 infants, 19 (24.6%) had neuroimaging abnormalities consistent with CZS. Among those, 9 (47.4%) had laboratory-confirmed ZIKV (via IgM ZIKA CSF). Out of the 9 laboratory-confirmed ZIKV, 8 (88.9%) of the mothers reported rash during pregnancy. Out of the infants with lab confirmation, 7 (77.8%) had CZS with microcephaly at birth, while 2 developed microcephaly postnatally.
Del Campo et al; 2017 [11]	Brazil	Infants with head circumference ≤33 cm	Case series	83 live-born infants, with findings on neuroimaging consistent with CZS	61/83 (73.5%) of the mothers reported more than 1 symptom—fever, rash, arthralgia, itch, conjunctival hyperaemia; 64 (77.1%) reported maculopapular rash. 57.4% (35/83) of the infants had severe microcephaly. ZIKV IgM in CSF was tested in 14 infants (12 were positive). Among 12 infants who were ZIKV lab-confirmed positive, 10 (83.3%) of the mothers reported rash.
Franca et al; 2016 [12]	Brazil	Liveborn infants with complete investigations from Brazilian MOH (microcephaly and CNS surveillance)	Case series	1501 newborn suspected cases, but 899 discarded; 602 cases for analysis	76 definite, 54 highly probable, 181 moderately probable, and 291 somewhat probable cases. Out of 76 definite cases (cases with lab-confirmed ZIKV), 71.4% of their mothers reported rash. Among the 76 definite cases, 13.2% had head circumference >–2SD. Among 319 definite or probable cases with full information, 161 (50%) had both microcephaly and a history of rash.
Leal et al; 2016 [13]	Brazil	Children born with microcephaly and lab-confirmed ZIKV	Case series	70 infants (0–10 mo) with microcephaly and lab evidence of ZIKV	Among the 70 infants with microcephaly and/or lab-confirmed ZIKV, 54 (86%) of their mothers reported rash during pregnancy. 43/70 (61.4%) of the infants had severe microcephaly.
Cuevas et al; 2016 [14]	Colombia	Reported cases of microcephaly	Case series	476 reported cases of microcephaly/147 infants had lab evidence of ZIKV infection by RT-PCR or immunohistochemistry	Among 476 infants and fetuses with microcephaly, a total of 306 (64%) were tested for ZIKA, and 147 (48%) had laboratory evidence of ZIKV. Among 476 infants with microcephaly, mothers of 164 (34%) reported symptoms compatible with ZIKV during pregnancy.
Van der Linden et al; 2016 [15]	Brazil	Infants with normal head size but lab-confirmed ZIKV who required clinical attention	Case series	13 infants with lab confirmation and normal head size	6/13 of the mothers reported rash between the second and fifth months of pregnancy. 6 infants had craniofacial disproportion (3 had redundant skin on the scalp at birth, and 3 infants had hip dysplasia). 11/13 infants had postnatal microcephaly, and all neuroimaging showed evidence of decreased brain volume.

Abbreviations: CNS, central nervous system; CSF, cerebrospinal fluid; CT, computed tomography; CZS, congenital Zika syndrome; IgM, immunoglobulin M; MOH, Ministry of Health; MR, magnetic resonance; NAT, nucleic acid testing; PRNT, plaque-reduction neutralization testing; RT-PCR, real-time polymerase chain reaction; ZIKV, Zika virus. ^a^These studies all report findings from the US pregnancy registry

The retrospective studies also consistently found that a substantial proportion of mothers of neonates with CZS reported no symptoms and so presumably had an asymptomatic prenatal ZIKV infection; however, the proportion of symptomatic vs asymptomatic women varied greatly between studies. This variation can partially be explained by the case definition: Some studies considered fever and at least 1 additional sign or symptom; in other studies, the case definition included only 1 symptom (usually rash). Another variation presented was the definition of the outcome. In some case series, all the mothers had laboratory-confirmed Zika infection during their pregnancy [9]; in other studies, the case definition was based on brain imaging consistent with ZIKV infection [11]. An additional explanation is recall bias and recruitment bias that would favor a history of rash or other symptoms compatible with ZIKV disease. In the retrospective studies, the proportion of CZS as a result of symptomatic maternal ZIKV infection ranged from 88.9% [10] to 34%, translating into a ratio of symptomatic-to-asymptomatic maternal infections between 5:1 and 1:2.

## CONCLUSIONS

This review documents that asymptomatic prenatal ZIKV infection can result in CZS. The retrospective studies (case-control and case series) showed a variable risk, and this variation reflects a combination of recruitment bias, recall bias, and varying case definitions. Prospective cohort studies are less affected by such bias, and the only published cohort studies to date are based on the US Zika Pregnancy and Infant Registry, which reported roughly similar numbers of CZS in neonates born to women with symptomatic and asymptomatic ZIKV infection.

Our findings have several implications. First, the ratio of asymptomatic-to-symptomatic infections in pregnancy appears to be lower in pregnant travelers returning to the United States compared with the population-based seroprevalence study on Yap Island, although recruitment bias toward symptomatic women may have played a role in the higher proportion of symptomatic infections seen in the US study. Second, it highlights that surveillance of women based on rash or other symptoms is not sufficient, and screening all pregnant women for ZIKV exposure is necessary in areas or countries where ZIKV is circulating. Taking into account that currently available diagnostics for ZIKV are suboptimal and hence may miss maternal ZIKV infections, birth defect surveillance for CZS needs to be strengthened. Third, given the low viremia levels, more sensitive diagnostic tools are urgently needed to improve maternal sceening. Fourth, as asymptomatic infections are likely associated with lower viremia, our findings suggest that even low levels of viremia could lead to CZS. A high bar is hence required for Zika vaccine development, possibly necessitating a vaccine that achieves complete reduction or prevention of viremia, for example, sterilizing immunity. The demonstration of a clinical benefit of a vaccine is usually based on a clinical end point. Our findings would justify selecting ZIKV infection rather than ZIKV disease as a clinical end point. However, the disadvantage of such an end point is the need for frequent sampling to detect asymptomatic infections, confounded by the limitations of current diagnostic assays. Perhaps protection against infection could be studied in at least a subset of an efficacy trial where the primary end point would be clinical ZIKV disease. Lastly, the ratio of asymptomatic-to-symptomatic infections was best described in the US cohort but needs to be confirmed by larger prospective cohort studies in endemic countries.
